# Proteomic Insight into the Molecular Function of the Vitreous

**DOI:** 10.1371/journal.pone.0127567

**Published:** 2015-05-28

**Authors:** Jessica M. Skeie, C. Nathaniel Roybal, Vinit B. Mahajan

**Affiliations:** 1 Omics Laboratory, Department of Ophthalmology and Visual Sciences, University of Iowa, Iowa City, IA, United States of America; 2 Department of Ophthalmology and Visual Sciences, University of Iowa, Iowa City, IA, United States of America; Pacific Northwest National Laboratory, UNITED STATES

## Abstract

The human vitreous contains primarily water, but also contains proteins which have yet to be fully characterized. To gain insight into the four vitreous substructures and their potential functions, we isolated and analyzed the vitreous protein profiles of three non-diseased human eyes. The four analyzed substructures were the anterior hyaloid, the vitreous cortex, the vitreous core, and the vitreous base. Proteins were separated by multidimensional liquid chromatography and identified by tandem mass spectrometry. Bioinformatics tools then extracted the expression profiles, signaling pathways, and interactomes unique to each tissue. From each substructure, a mean of 2,062 unique proteins were identified, with many being differentially expressed in a specific substructure: 278 proteins were unique to the anterior hyaloid, 322 to the vitreous cortex, 128 to the vitreous base, and 136 to the vitreous core. When the identified proteins were organized according to relevant functional pathways and networks, key patterns appeared. The blood coagulation pathway and extracellular matrix turnover networks were highly represented. Oxidative stress regulation and energy metabolism proteins were distributed throughout the vitreous. Immune functions were represented by high levels of immunoglobulin, the complement pathway, damage-associated molecular patterns (DAMPs), and evolutionarily conserved antimicrobial proteins. The majority of vitreous proteins detected were intracellular proteins, some of which originate from the retina, including rhodopsin (RHO), phosphodiesterase 6 (PDE6), and glial fibrillary acidic protein (GFAP). This comprehensive analysis uncovers a picture of the vitreous as a biologically active tissue, where proteins localize to distinct substructures to protect the intraocular tissues from infection, oxidative stress, and energy disequilibrium. It also reveals the retina as a potential source of inflammatory mediators. The vitreous proteome catalogues the dynamic interactions between the vitreous and surrounding tissues. It therefore could be an indirect and effective method for surveying vitreoretinal disease for specific biomarkers.

## Introduction

The vitreous is an optically transparent extracellular matrix that coats the retina, ciliary body, and lens.[1] The vitreous fills approximately 80% of the inner eye and is over 98% water.[2, 3] The remaining 2% contains proteins, polysaccharides, proteoglycans, and metabolites, but the physiologic function of this fraction is largely unknown.[4] In contrast, the pathological function of the vitreous is apparent in several retinal diseases.[2] Vitreoretinal traction, for example, underlies a number of acquired conditions that include retinal detachment, epiretinal membrane, proliferative diabetic retinopathy, macular hole, and proliferative vitreoretinopathy. Age-related vitreous degeneration causes visually significant opacities. Because mutations in vitreous collagens can lead to abnormal eye size and vitreoretinal degeneration,[5] a few congenital vitreoretinopathies provide some molecular insight. Surgical removal of the vitreous can be effective in managing many of these conditions, but the underlying molecular mechanisms remain poorly understood. The developmental and disease roles of the vitreous emphasize its structural function, but extracellular matrices have important biological functions that are revealed when the molecular components are known.

To better understand the function of a tissue, gene expression analysis is a common approach for cataloguing molecular components. The vitreous, however, is a complex extracellular tissue where proteins originate from local hyalocytes and tissues, some of which are local and others are outside the eye. The vitreous proteome becomes drastically altered as a result of systemic disease. Two well-documented cases are an altered vitreous proteome in proliferative diabetic retinopathy and the vitreous deposition of amyloid in systemic amyloidosis.[6–12] Therefore, gene expression analysis of local eye tissues or the few resident hyalocyte cells could fail to account for many vitreous proteins in normal and disease states. Proteomic analysis circumvents this pitfall by identifying every expressed protein within a tissue, regardless of its site of synthesis. The human and equine vitreous has been subjected to multiple fractionation techniques and mass spectrometry analysis in the normal and disease state studies.[8, 13, 14] The proteome of the normal mouse vitreous was recently published, [15] and suggested the molecular findings could be translated using animal models of vitreous disease.[15] For example, proteomic studies identified a number of proteins that distinguish the normal from diseased vitreous in subjects with diabetic retinopathy, age-related macular degeneration, proliferative vitreoretinopathy, and uveitis.[13, 14, 16] These studies were recently reviewed by Angi et. al.[17] One limitation of existing vitreous proteomic studies, however, has been the sampling method.[18] Surgical biopsies only collect proteins from the vitreous core, so other vitreous substructures, such as the vitreous base and cortex, have not been studied. Many vitreoretinal diseases localize to these specific vitreous substructures, so it is important to determine the normal proteomes of these anatomical regions.

**Fig 1:**
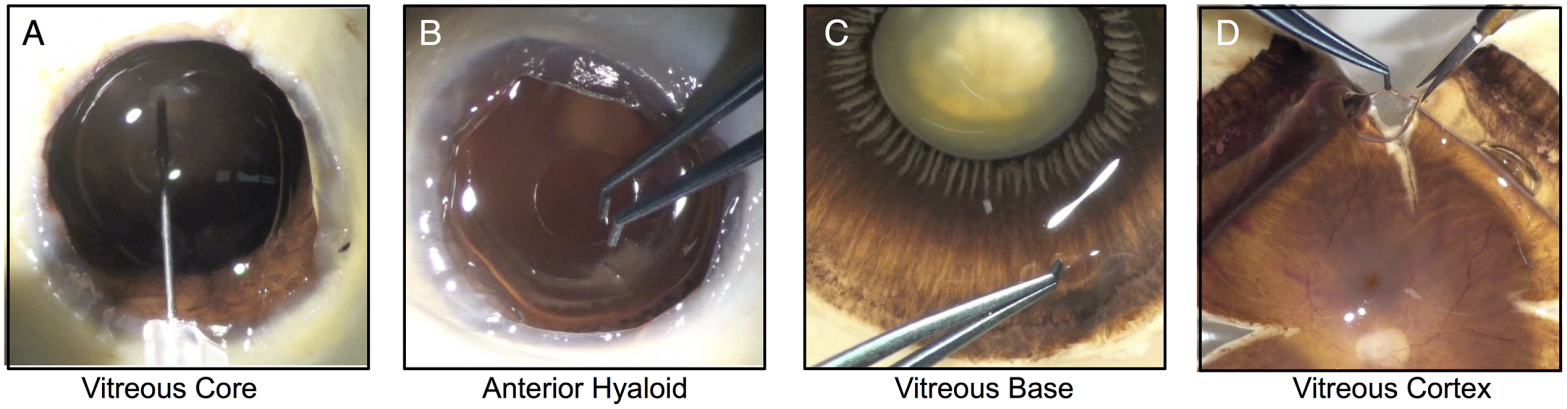
Human vitreous component dissection images. The images are shown in the order they are dissected. A. The vitreous core is aspirated using a 23-gauge needle following the removal of the anterior portion of the eye. B. After the vitreous core is aspirated, the anterior hyaloid becomes visible as a translucent ring. This tissue can be grasped with Colibri forceps, cut, and collected. C. The vitreous base is a thick, viscous tissue lying over the ora serrata. This image is for visualization purposes (still containing the anterior iris and lens) to show the pars plicatta, pars plana, ciliary body, and ora serrata. The vitreous base is grasped using Colibri forceps, pulled away from the ora serrata, and cut with Vannas scissors. D. The vitreous cortex is collected by cutting the posterior pole into quadrants and grasping between two sections. It is lifted and cut away from the posterior pole.

The human vitreous can be divided into four distinct anatomical regions (Fig 1). The vitreous core is the largest compartment and fills the center of the eye. The vitreous cortex forms a shell around the vitreous core and forms loose connections to the adjacent retina. The vitreous base anchors the vitreous and cannot be fully removed as it is firmly adherent to the ciliary body and anterior retina. The anterior hyaloid extends from the ciliary body to the lens.[2] The anatomical locations, differing biomechanical properties, and disease localization associated with these vitreous regions suggest functional differences.[18] The proteome of the four distinct vitreous substructures of the human vitreous has yet to be described. To address this issue, our lab developed a method to dissect each substructure from recent postmortem specimens.[19] In this study we examined the differential protein composition of the four substructures that comprise the human vitreous.

## Methods

### Human Vitreous Tissue Collection

**Fig 2:**
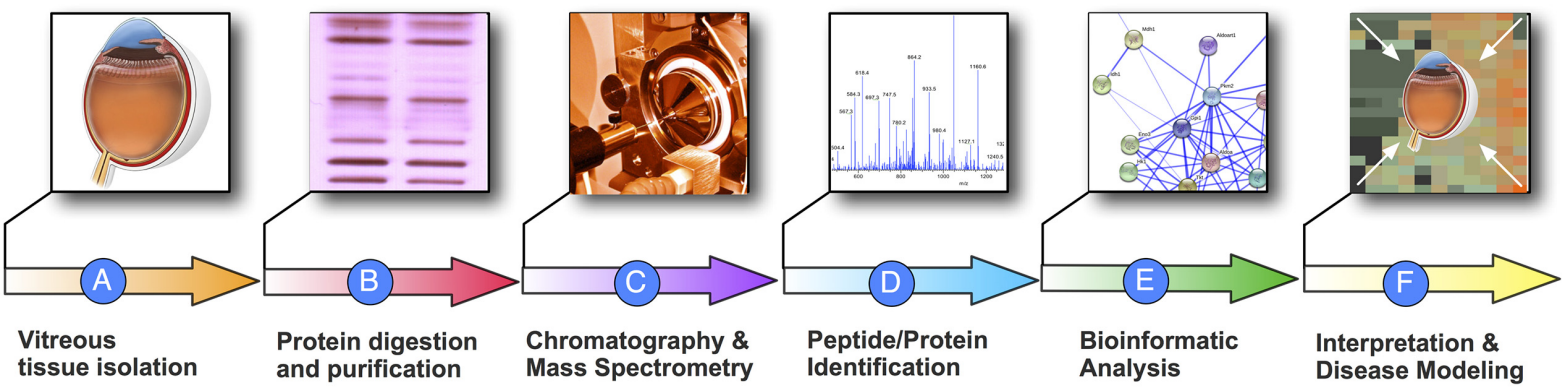
Vitreous proteome analysis pipeline. A. The human vitreous was dissected into core, anterior hyaloid, cortex, and base. B. Fractions of proteins were isolated and digested. C. The peptide fragments were analyzed using multi-dimensional LC-MS/MS. D. X!Hunter, X!!Tandem, and OMSSA were used for peptide fragment identification. E. Proteins were further analyzed using bioinformatics software. F. Interpretation of the protein datasets in reference to disease biology.

This study was approved by the University of Iowa’s Institutional Review Board and adheres to the Declaration of Helsinki. Human donor tissue was obtained from the Iowa Lions Eye Bank, Iowa City, IA within 5 hours of death. Three eyes from healthy donors were used in this study. They were obtained from a 68 year old male, an 87 year old female, and a 60 year old female. None of the eyes had a history of or showed any gross signs of disease including pathologic myopia, diabetic retinopathy or other vitreoretinal diseases. Vitreous samples were collected as previously described, [19] without any evidence of cellular contamination. Briefly, within five hours of patient expiration, the anterior portion of the eye was removed and the vitreous core was aspirated. The anterior hyaloid was cut circumferentially using Vannas scissors and Colibri forceps. The eye was then flowered into quadrants. The vitreous base was dissected away from the ora serrata and the posterior hyaloid was collected from the posterior pole by pulling it away from the retina (Fig 1). Vitreous samples were flash frozen in liquid nitrogen and stored in our biorepository until processed for mass spectrometry (Fig 2A).[20]

### Multidimensional Protein Identification Technology (MudPIT) Mass Spectrometry

Mass spectrometry was performed as previously described.[15] Four vitreous substructures were obtained from each of three eyes, and the twelve samples were analyzed independently by mass spectrometry. Each substructure was analyzed in triplicate for statistics and bioinformatics. Proteins were prepared for digestion using the filter-assisted sample preparation (FASP) method (Fig 2B and 2C).[21] Concentrations of proteins were measured using a Qubit fluorometer (Invitrogen). Peptides were desalted using C_18_ stop-and-go extraction (STAGE) tips.[22] Peptides were fractionated by strong anion exchange STAGE tip chromatography. Fractions were analyzed by liquid chromatography tandem mass spectrometry (LC-MS/MS) (Fig 2C).

Liquid chromatography was performed on an Agilent 1100 Nano-flow system where mobile phase A was 94.5% MilliQ water, 5% acetonitrile, 0.5% acetic acid and mobile phase B was 80% acetonitrile, 19.5% MilliQ water, 0.5% acetic acid. Trap and analytical columns were packed with 3.5 um C_18_ resin (Zorbax SB, Agilent) and the LC was interfaced with a dual pressure linear ion trap mass spectrometer (LTQ Velos, Thermo Fisher) using nano-electrospray ionization. An electrospray voltage of 1.8 kV was applied to a pre-column tee and the mass spectrometer acquired tandem mass spectra from the top 15 ions in the full scan from 400–1400 m/z. Dynamic exclusion was set to 30 seconds.

Mass spectrometer output RAW data files were converted to MGF format using msconvert and all searches required strict tryptic cleavage, 0 or 1 missed cleavages, fixed modification of cysteine alkylation, variable modification of methionine oxidation and expectation value scores of 0.01 or lower. The MGF files were searched with X!Hunter[23] against the latest library available in 2010 on the GPM[24] and X!!Tandem[25, 26] using both the native and k-score[27] algorithms as well as by OMSSA.[28] All searches were performed on Amazon Web Services-based cluster compute instances using the Proteome Cluster interface. XML output files were parsed and non-redundant protein sets were determined (Fig 2D). Proteins were required to have 2 or more unique peptides with E-value scores of 0.01 or less. Relative quantitation was performed by spectral counting. Data were normalized based on total spectral counts (hits) per sample.

### Bioinformatic and Statistical Analysis

Proteins were considered identified if they had an expectation value < 0.01 (less then 1% chance of being a random assignment). Bioinformatic analyses were used to determine significant protein expression (Partek Geonomics Suite 6.6), gene ontology (GO terminology, Panther 7.2), and pathway representation (MetaCore) (Fig 2E). Using Partek Genomics Suite 6.6, the protein lists for all three mass spectrometry runs for all regions of choroid-RPE tissue were analyzed. All data values were set to a minimum of 0.001, normalized to log base 2, and compared using ANOVA. Statistically significant proteins (p < 0.05) were visualized using an un-discriminated clustered heatmap with a normalized clustering function. Some proteins that showed a trend but did not meet statistical significance were analyzed further because they were an important component of a well-represented pathway or classification.

Pie charts were created for the visualization of Gene Ontology GO distributions within the list of proteins using Panther 7.2 Classification system under the Batch ID search menu. Pie charts were created for each GO term category including biological process, molecular function, and cellular component.

MetaCore (GeneGO Inc., St. Joseph, MI, USA) OMICs data analysis was used to determine the most significant protein signaling/interaction pathways. MetaCore generates protein pathway maps using curated literature databases.[29] The names of pathways are representative of single proteins in that pathway, known signaling cascades, or even an associated disease, and therefore may not coincide directly with our dataset even though our dataset contained several proteins within the pathway. Information regarding MetaCore software can be obtained at www.genego.com. Lists of vitreous proteins were curated using Excel and uploaded into the MetaCore website. Pathway lists were created comparing regional protein lists simultaneously, i.e. differentially expressed vitreous protein lists were analyzed simultaneously. Lists of the 50 most significant pathways were exported to excel files. Venn diagram was created using Venny online software (Venny, http://bioinfogp.cnb.csic.es/tools/venny/) (Fig 2F).[30]

## Results

### Mass Spectrometry Overview

Vitreous samples underwent trypsinization and multidimensional liquid chromatography before analysis by tandem mass spectrometry. In the anterior hyaloid, we identified 2,079 unique proteins (33,528 spectra with 3,204 unique peptides). In the vitreous cortex, there were 2,440 unique proteins (81,295 spectra with 4,878 unique peptides). The vitreous base had 2,117 unique proteins (68,833 spectra with 4,647 unique peptides), and the vitreous core had 1,612 unique proteins (105,255 spectra with 4,093 unique peptides). The most abundant proteins were transferrin, albumin, clusterin, serpin peptidase inhibitors, transthyretin, crystallins, fibrillin 1, vimentin, immunoglobulins, enolase, C3, C4A, C4B, ceruloplasmin, opticin, and pyruvate kinase. The total spectra for the four samples on average had a standard deviation of 29,901, which is 41.4% of the average total. This indicates that each of the vitreous substructures varied from one another in complexity and could be identified as distinct tissues based on their molecular signature.

**Fig 3:**
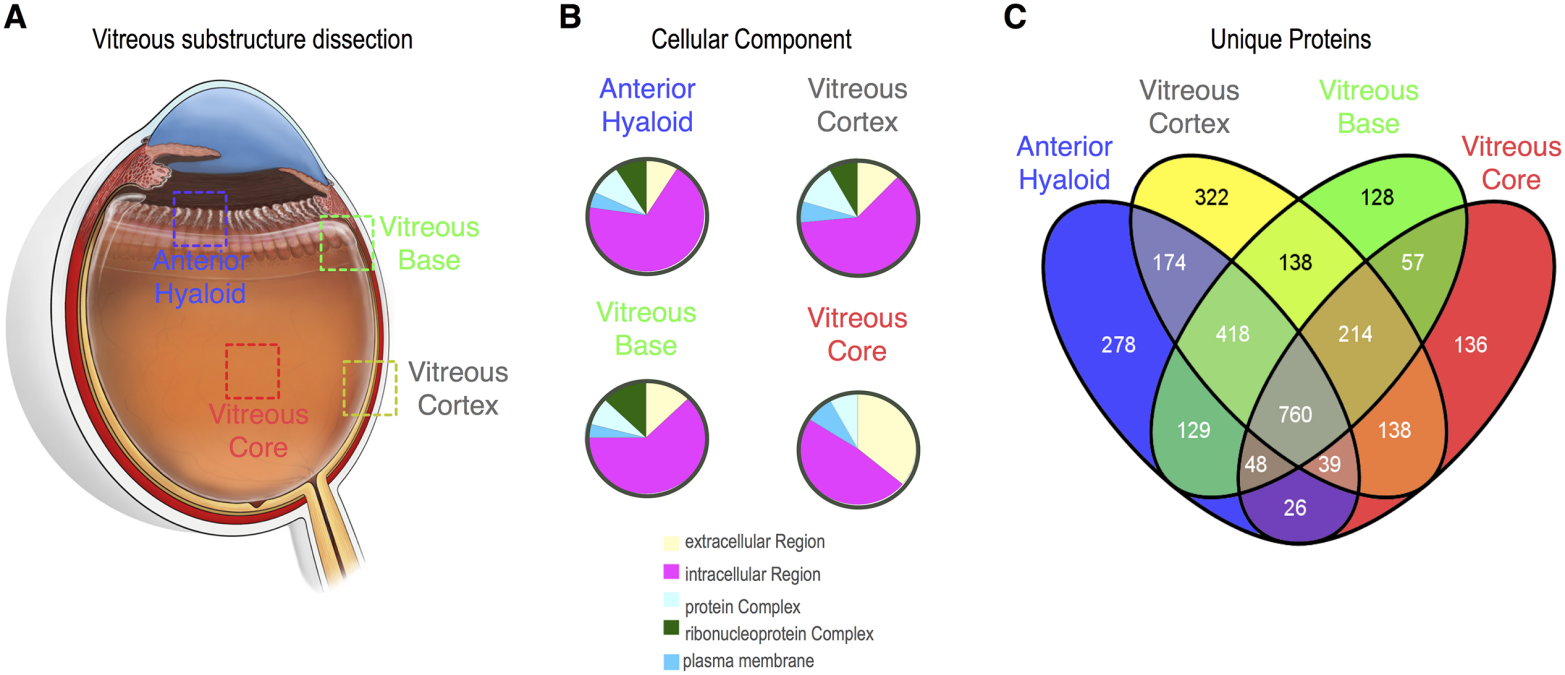
Global analysis of human vitreous substructures. A. Cross section of human eye depicts the four regions of vitreous tissue collected and includes the anterior hyaloid, vitreous base, vitreous cortex, and vitreous core. B. Gene ontology of the four distinct vitreous regions shows that within the cellular component category, most proteins are intracellular. C. Venn diagram shows the number of unique and overlapping proteins identified in each of human vitreous substructure.

Vitreous substructures display different physical properties that suggest distinct molecular profiles. To identify proteins unique to each structure, a comparative analysis was performed (Fig 3). There were 278 unique proteins in the anterior hyaloid, 322 in the vitreous cortex, 128 in the vitreous base, and 136 in the vitreous core (Fig 3B and 3C). These proteins underwent further analysis described below.

### Gene Ontology

**Fig 4:**
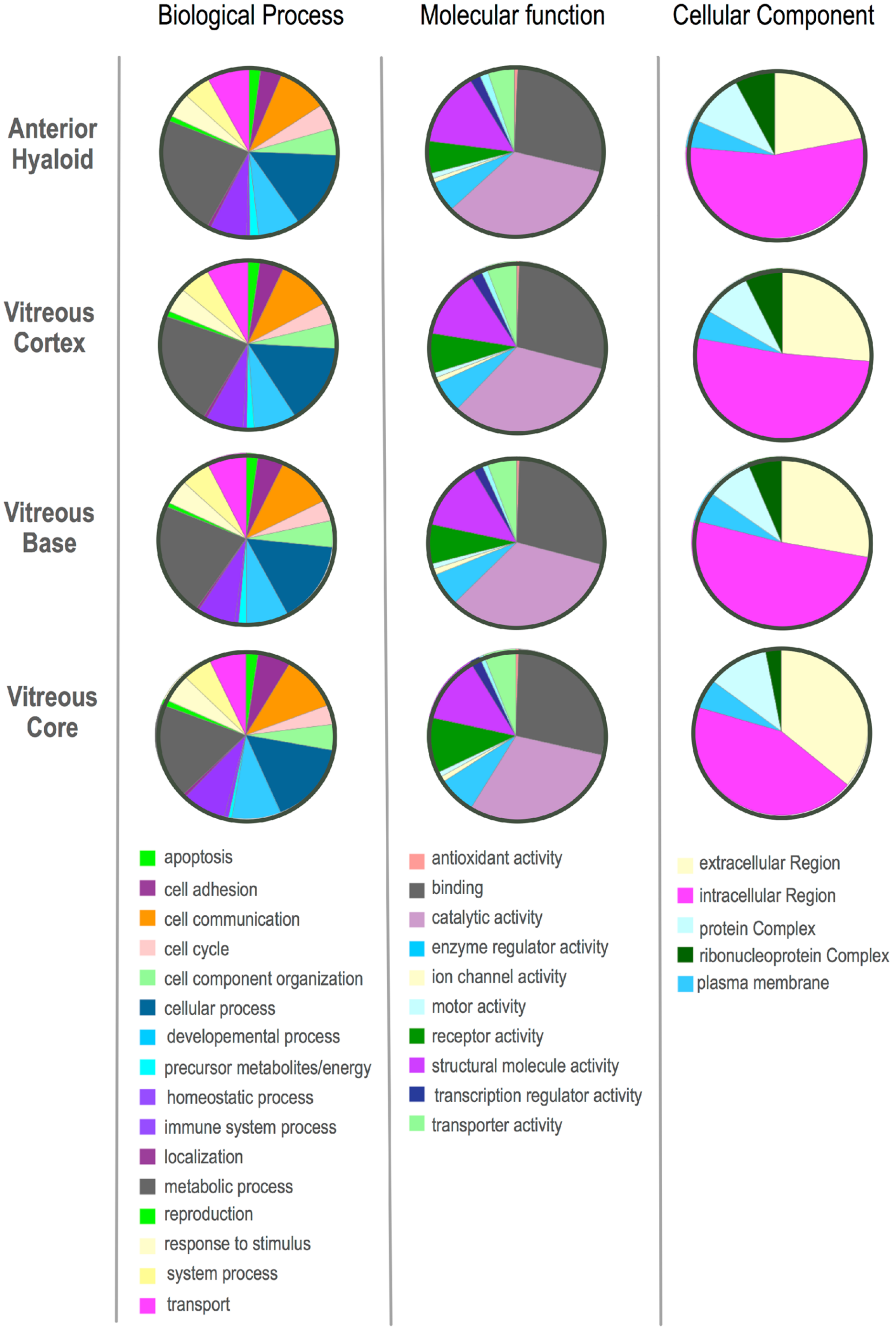
Gene ontology (GO) distributions and pathway analysis of human vitreous proteins show tissue similarity. Expressed proteins of the anterior hyaloid, vitreous cortex, vitreous base, and vitreous core. Proteins were grouped into sub-categories of biological processes, molecular functions, and cellular component for each of the four regions.

To obtain a global view of the biological processes, molecular function, and cellular components represented in the four distinct vitreous compartments, a gene ontology analysis was performed. When comparing the total protein profiles for the four regions, the gene ontology summaries were similar. The highest represented categories were metabolic process, binding, catalytic activity, and intracellular regions (Fig 4). Since the vitreous does not contain cells, it was expected that the proteins would be extracellular. Surprisingly, about half of the proteins were intracellular and highest in the vitreous core, suggesting the surrounding tissues were releasing intracellular proteins into the otherwise acellular vitreous. The intracellular proteins could be degradation products from cellular tissues surrounding the vitreous, or could be functional units transported in the vitreous through microvesicles. Which has been shown in the aqueous and other fluid tissue.[31]

**Fig 5:**
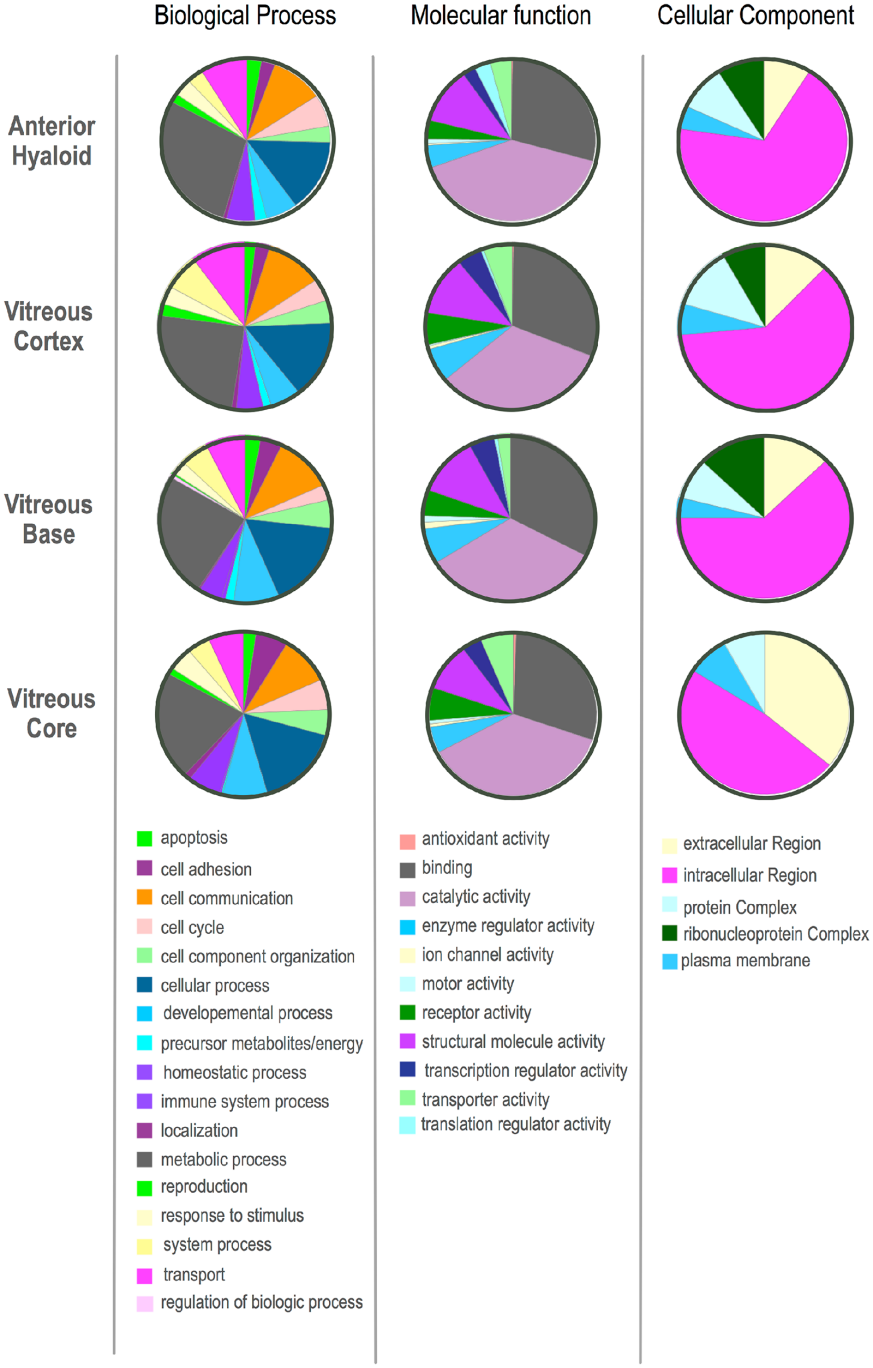
Gene ontology (GO) distributions and pathway analysis of human vitreous proteins show tissue differences. Expressed proteins of the anterior hyaloid, vitreous cortex, vitreous base, and vitreous core. Proteins were grouped into sub-categories of biological processes, molecular functions, and cellular component. These proteins are unique to each region of the vitreous as determined using the Venn diagram in Fig 3C.

After identification of differentially expressed proteins in the substructures (Fig 3C), differences in gene ontology (GO) categorization emerged (Fig 5). The vitreous core had more proteins in the cell adhesion category than the other three tissues. The anterior hyaloid and vitreous base had precursors to metabolites, whereas the core did not. The vitreous base was the only tissue with proteins in the regulation of biologic process category. In molecular function classifications, the core had more antioxidant proteins than the other tissues, while it was missing translation regulatory proteins. In the cellular component category, every tissue had similar representation, except there were no ribonucleoprotein complex proteins in the vitreous core (Figs 3B and 5). Together, this indicated that each substructure expresses distinct functional categories of proteins not associated with mechanical properties of a tissue.

### Molecular Pathways

**Fig 6:**
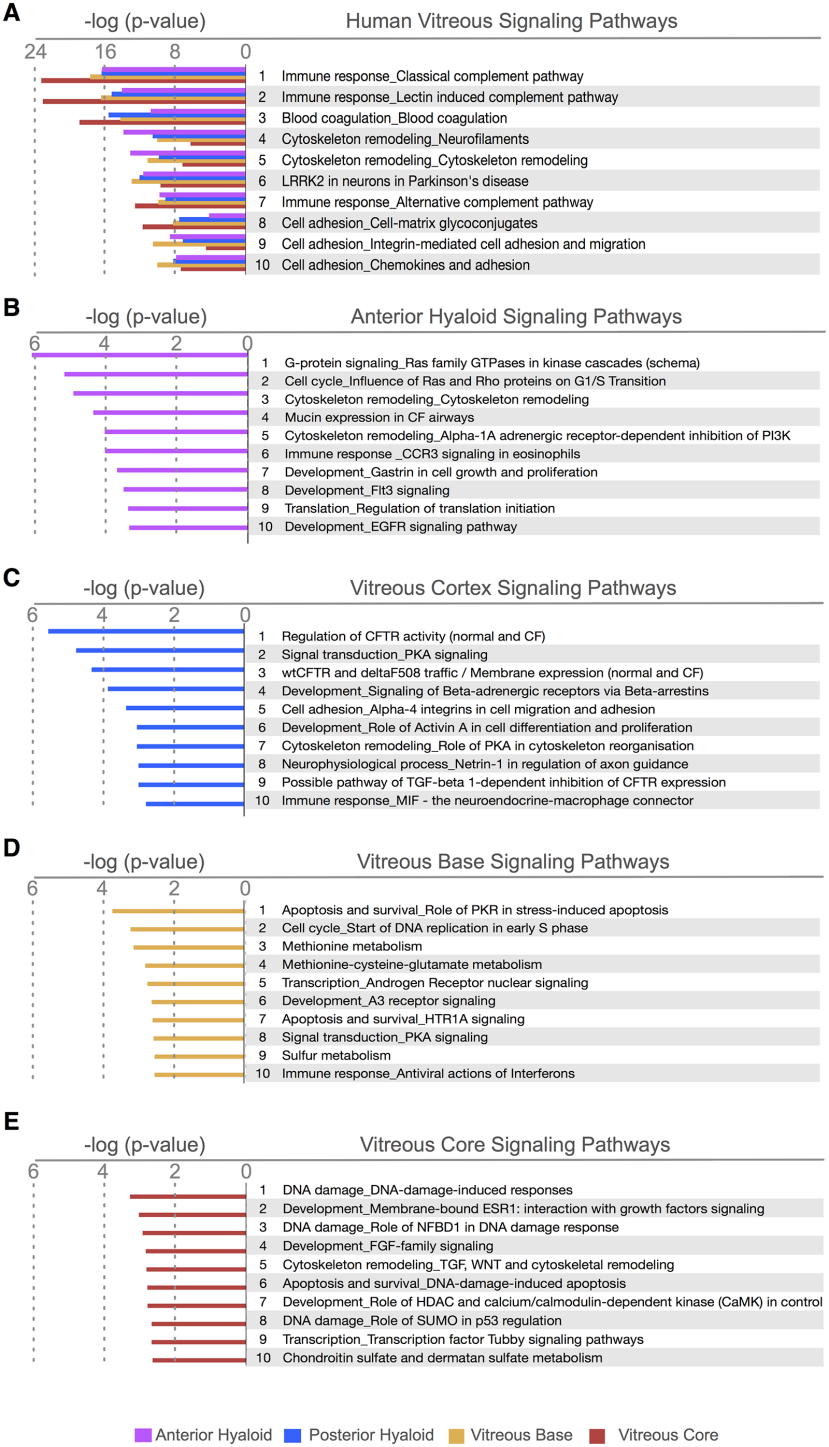
Proteome pathway analysis of human vitreous substructures. A. Top ten pathways represented in all four of the vitreous regions. The relative abundance of proteins in each pathway is shown for each tissue. Not all proteins represented in one substructure are the same as another. B, C, D, E. The top ten pathways based on uniquely expressed proteins are listed for each substructure: (B) anterior hyaloid, (C) vitreous cortex, (D) vitreous base, and (E) vitreous core.

A molecular pathway analysis identified groups of functionally linked proteins. Analyzing the four substructures together, the top pathways were the classical, alternative and lectin induced complement cascade immune responses, blood coagulation, cytoskeleton remodeling, leucine-rich repeat kinase 2 (LRRK2) in neurons, and cell adhesion (Fig 6A).

Next, from the list of differentially expressed proteins (Fig 3C), highly represented pathways were determined. The top pathways in the anterior hyaloid included CCR3 signaling immune response, Flt3 signaling, and EGFR signaling (Fig 6). In the vitreous cortex, the most represented pathways were regulation of CFTR activity, PKA signaling, activin A in cell differentiation and proliferation, TGF-beta 1-dependant inhibition of CFTR expression, and MIF mediated immune response (Fig 6C). The pathways in the vitreous base were PKR in apoptosis, DNA replication, methionine-cysteine-glutamate metabolism, HTRA1 signaling, PKA signaling, sulfur metabolism, and interferon immune response (Fig 6D). The vitreous core had the highest representation of proteins in the DNA damage (NFBD1 or SUMO regulated), and development (ESR1, FGF-family, or HDAC signaling) pathways (Fig 6E). Again, we found that intracellular protein pathways were highly represented and showed distinct signatures between the vitreous substructures.

### Protein Interaction Networks

When the entire proteome was analyzed for interaction networks, the vitreous cortex had 2,450 nodes (proteins) and 4,981 edges (interactions), the anterior hyaloid had 2,085 nodes and 3,576 edges, the vitreous base had 2,083 nodes and 3,816 edges, and the vitreous core had 1,574 nodes and 2,486 edges. Interactions represent established binding, signaling, structural, activation, or inhibition activity between two proteins.

**Fig 7:**
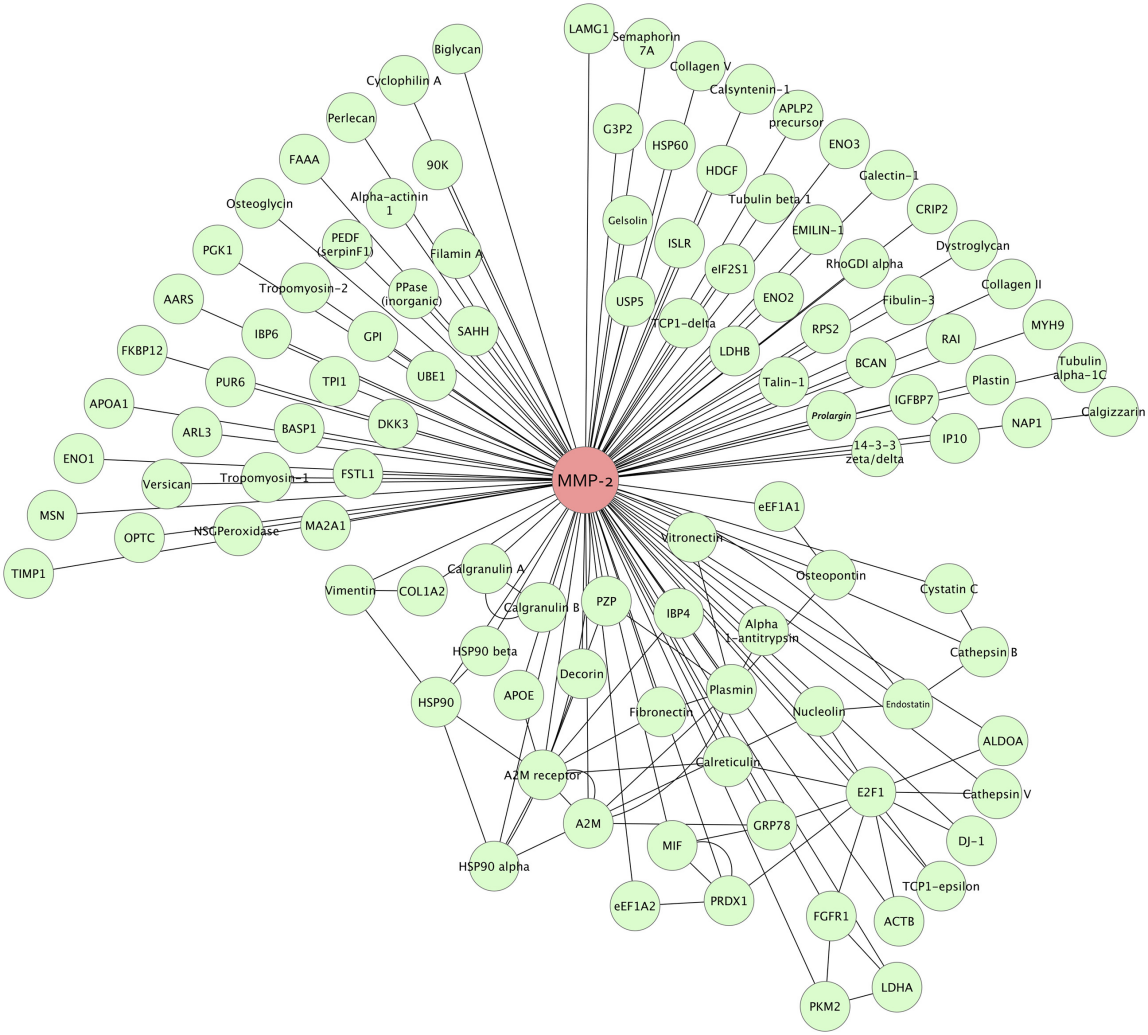
Matrix metalloproteinase 2 (MMP-2) network. MMP2 is the largest network common to all four regions of the human vitreous humor. MMP-2 is involved in the proteolysis of extracellular matrix proteins, and 108 targets were found in the vitreous.

The largest network among all four substructures was the MMP-2 network (Fig 7). There were 108 targets of MMP-2 expressed in the vitreous. MMP-2 breaks down extracellular matrix proteins during development and disease. Matrix metalloproteinases in the eye are responsible for tissue turnover events including wound healing, neovascularization, vitreous liquefaction, nerve damage, and cell degeneration.[32] Our findings indicate MMP2 is a major regulator of vitreous dynamics.

**Fig 8:**
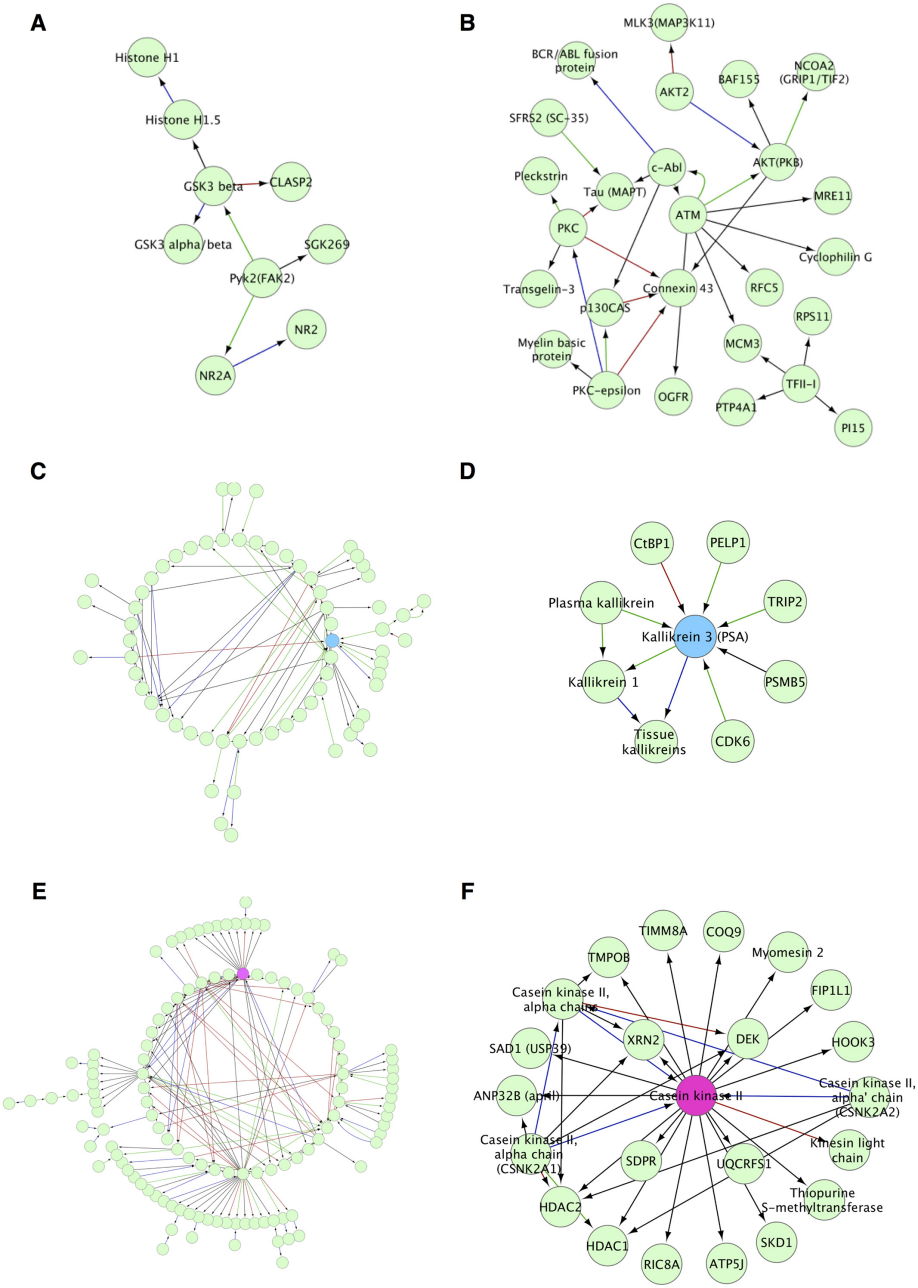
Network analysis reveals the largest unique protein networks for vitreous substructures. A. The largest hub in the vitreous base was the GSK3 beta hub. B. In the vitreous core, the largest unique network is the ATM network. C, D. The largest hub in the anterior hyaloid is the kallikrein 3 (PSA) network. E, F. The largest network in the vitreous cortex is casein kinase II.

The vitreous base and vitreous core showed low complexity protein networks. The largest network unique to the vitreous base was the glycogen synthase kinase 3 beta subunit (GSK3 beta) protein complex (Fig 8). This kinase is involved metabolism and transcription through Wnt/β-catenin signaling.[33, 34] The Ataxia Telangiectasia Mutated (ATM) and protein kinase C (PKC) interactomes were unique to the vitreous core (Fig 8B). These proteins are involved in DNA repair and cellular signaling, respectively.[35, 36]

The networks in the anterior and vitreous cortex were highly complex. Subsets of these networks were chosen for visualization purposes. A subset of proteins from the anterior hyaloid interact with kallikrein 3 (Fig 8C and 8D), and a subset in the vitreous cortex interact with casein kinase II (Fig 8E and 8F). Kallikreins are serine proteases with a variety of functions and serve as a potential biomarkers.[37] Casein kinase II regulates cellular processes including metabolism, transcription, translation, cell replication, and signal processes.[38] Some of these network proteins were relatively low abundance, but their networks were well represented.

### Regional Protein Expression

**Fig 9:**
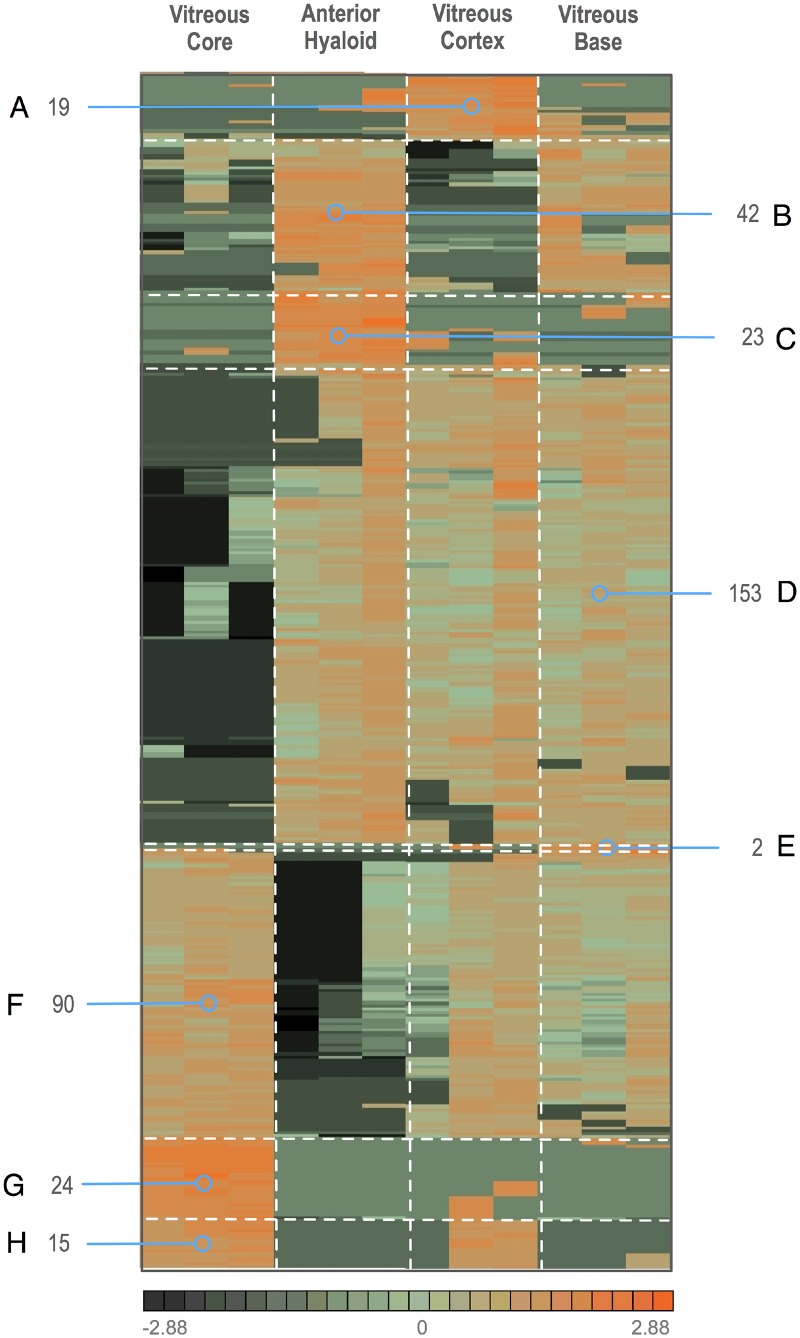
Unbiased clustering of differentially expressed proteins (p<0.05) in the four human vitreous substructures. Proteins represented in this cluster analysis were determined by ANOVA, p<0.05. The heatmap is divided into regions: A. proteins highest in the vitreous cortex, B. proteins highest in the anterior hyaloid and the vitreous base, C. proteins highest in the anterior hyaloid, D. proteins absent or low in the vitreous core but represented in the other three tissues, E. proteins highest in the vitreous base, F. proteins in all tissues except the anterior hyaloid, G. proteins unique to the vitreous core, and H. proteins highest in the vitreous core and vitreous cortex.

We compared the four distinct regions of the vitreous to determine unique (Fig 1C) and differentially expressed proteins (Fig 9). Regionally expressed proteins in our normal eyes create a good baseline for future disease proteomics studies.

### Extracellular Matrix Proteins

Extracellular matrix proteins were regionally distributed. The vitreous base was the only tissue containing collagen 28A1 and the vitreous core contained both collagen 4A1 and 4A4. IGLV3-10 is an extracellular protein that provides structure and protein sequestering in the extracellular matrix. It was found in the vitreous base and cortex. Proteoglycans can affect tissue adhesiveness. These included proteoglycan 4, found in the anterior hyaloid and vitreous base only; sparc/osteonectin 2 (SPOCK2) in the vitreous cortex and core; N-glycanase 1 in the cortex only, and elastin in only the vitreous core and base. The regional specificity of extracellular matrix proteins could account for surgical differences in the mechanical properties.

### Proteases

Numerous proteases and protease pathways were found in the vitreous. Network analysis showed that MMP2 was the major protease network of the vitreous. We also found MMP8, MMP9, and MMP28. Cathepsin C (CTSC), a cysteine proteinase involved in inflammation, was found only in the vitreous core and base. Cystatin C (CST3), an extracellular cysteine protease inhibitor, was highly expressed in the core.

### Coagulation Cascade

Thirty-nine components of the coagulation protease cascade were identified. Activation of the coagulation cascade triggers the formation of blood clots and signals cells.[39] Fibrin is formed by the conversion of fibrinogen by thrombin, and fibrin rapidly forms around vitreous foreign bodies, for example. Thrombin (factor II) was found in all components of the vitreous. Fibrinogen components A, B, and G were all also found in all substructures. In contrast, factors XIIIa1 and XIIIb were only found in the vitreous cortex and base. Protein C (PROC) is a vitamin K dependent glycoprotein found in the plasma and is an inhibitor of the blood coagulation cascade. It was only found in the vitreous core and cortex. Serpin peptidase inhibitor (SERPINE2) is a serine protease inhibitor for thrombin, plasmin and trypsin. It was only found in the vitreous core and cortex.

### Complement Cascade

The high level and number of complement proteins found in the vitreous indicates an important role for the innate immune system. We found a total of 29 complement cascade proteins, including all of the proteins that comprise the membrane attack complex (MAC; C5b-9). Most complement proteins were present in all four vitreous substructures. Inhibitor of compliment activation, complement factor D (CFD) was found in every substructure. Other complement factors showed regional expression. C1RL was only detected in the vitreous cortex and core. MASP-1 and MASP-2 were only in the vitreous core, cortex, and base. CFHR3 was found in every substructure, except the anterior hyaloid. CFHR5 was found in every substructure, except the core. Complement component 1, q subunit, A chain (CIQA) was only found in the vitreous core and cortex. The high representation of the complement cascade suggests it may have a broad pathophysiological role in ocular disease not limited age-related macular degeneration.[40]

### Angiogenic Proteins

Angiogenin (ANG), a promoter of angiogenesis, [41] was found in the vitreous core and cortex. Angiopoietin-like 1 (ANGPTL1) was unique to the vitreous core and cortex. Angiopoietins are proteins known for their role in angiogenesis, being both stimulatory and inhibitory.[42] Epidermal growth factor (EGF), which is a mitogen that increases cell proliferation and differentiation,[43, 44] was unique to the anterior hyaloid and vitreous base. There were low levels of several FGF cytokines, but the angiogenic cytokines VEGF and PDGF were absent.

### Cellular Signaling

Mitogen-activated protein kinase 1 (MAPK1) and mitogen-activated protein kinase 3 (MAPK3) transmit intracellular signals involved in cell proliferation, transcription, development, and apoptosis in response to extracellular signals. These proteins were found in the anterior hyaloid, vitreous cortex and vitreous base. Mitogen activated protein kinase kinase kinase kinase 4 (MAP4K4), which is involved in mediating TNF-alpha activity through the activation of MAPK8, was unique to the anterior hyaloid and vitreous base. Acid phosphatase 1, soluble (ACP1) was found only in the anterior hyaloid. ACP1 is both an acid phosphatase and a tyrosine phosphatase. Protein phosphatase 2, catalytic subunit, alpha isozyme (PPP2CA) was found in the vitreous cortex and base. PPP2CA is a member of the Ser/Thr phosphatase family and an inhibitor of cell growth and division. The differential expression of cellular signaling proteins may reflect different regulation of cellular changes in nearby tissues.

### Biomarkers for Vitreoretinal Disease

Several retinal proteins were also found in the vitreous with highest amounts of peptides found in the posterior cortex. These proteins included, rhodopsin (RHO), glial fibrillary acidic protein (GFAP), and phosphodiesterase 6 (PDE6). Low counts of GFAP and PDE6 peptides were also present in the vitreous core. Interestingly, retinal pigment epithelium-specific protein 65kDa (RPE65) was identified in the vitreous cortex. RPE65 produces 11-cis retinal and mutations in RPE65 are associated with Leber congenital amaurosis type 2 (LCA2) and retinitis pigmentosa. This indicates that the normal vitreous contains proteins arising from the outer retina and RPE, and these proteins could be significantly elevated in retinal or RPE degenerative diseases.

### DAMPs

The vitreous is a key site of inflammation in posterior uveitis, but resident factors have not been determined. Damage-associated molecular patterns (DAMPs) are endogenous, non-infectious proteins that can cause inflammation and/or tissue damage following their release during tissue injury.[45] We found 46 known DAMPs in the human vitreous, including several heat shock proteins (HSPs), S100 proteins, HMGB1, hyaluronan-like proteins, versican, heparin sulphate proteoglycan, and biglycan. During infection, inflammation or trauma, breakdown of these components could further activate inflammatory pathways as was found in autoimmunity, diabetes, aging, mouse models of retinal degeneration, and Alzheimer’s disease.[45–49]

## Discussion

Proteomic analysis using multidimensional liquid chromatography and tandem mass spectrometry is a powerful method to profile proteins rather than genes. It is particularly important when studying complex extracellular tissues, such as the vitreous, where both local and remote tissues contribute to the protein composition. The purpose of our study was to create a reference dataset for the vitreous proteome that highlights its unique substructures. Since surgical and clinical disease perspectives emphasize vitreous mechanics, there is an expectation and bias towards structural proteins. Instead, using an unbiased omics approach, we found many other protein categories.

Prior studies have applied proteomics to surgical vitreous core biopsies [8, 13, 14]. This study analyzes the vitreous substructure proteomes from fresh post-mortem eyes. This has the potential to capture the proteomic diversity of the vitreous substructures and their complex relationships with adjacent tissues and the hematologic system.

We identified thousands of proteins, many of which were unique to a particular vitreous substructure. Bioinformatic analysis showed that the proteins were not randomly assorted, but could be organized into functional pathways. This indicates that the vitreous functions to control infection, regulate oxidative stress, and metabolize energy. Although the eye is immune privileged, the innate (complement) and adaptive (immunoglobulin) immune systems are highly represented. Protease signaling, including the blood coagulation cascade and matrix metalloproteases, is also a major feature of the vitreous. Together, this knowledge should help link specific proteins and protein pathways to pathophysiological processes, such as age-related vitreous degeneration, cataract formation after vitrectomy, fibrosis in proliferative vitreoretinopathy diabetic retinopathy, and inflammation in endophthalmitis and uveitis.[50] The observation that vitrectomy may improve uveitis,[51] for example, may be due to the removal of DAMPs.[45, 51–54]

Interestingly, the majority of proteins found in the vitreous were intracellular. This finding is supported by several previous studies in humans and our findings in the mouse vitreous, where there are no cellular contaminants.[8, 11, 15] While some protein products may represent degraded proteins, others are likely to have normal functions. Numerous glycolysis and gluconeogenesis proteins were identified in the vitreous, and they are carried by microvesicles to regulate glucose, ATP, and pH in the extracellular space.[31] Microvesicles are also released during cellular activation and are an indicator of disease pathology.[31] The role of microvesicles and their protein constituents has yet to be explored in the vitreous, but biomarkers for age-related macular degeneration have been identified in microvesicles from aqueous chamber fluid.[55]

Since the collection the young, non-diseased human eye tissues is extraordinarily rare and frequently not approved by institutional review boards, the present study utilizes human vitreous from older individuals, and the findings may not extend to younger eyes. As the human vitreous ages it undergoes progressive liquefaction. The macromolecular composition and viscosity of the vitreous is influenced by age, state of the lens and the presence of vitreous and retinal pathology. The age range of the patients (60–87) in the current study most likely influenced the vitreous protein content to reflect age-related changes in the vitreous. Proteins and signaling pathways specific to younger patients and those with specific vitreo-retinal diseases will need to be individually studied in the future but can still be compared to the human vitreous protein library repository described herein.

Proteomic insights indicate the vitreous is a physiologically active, complex tissue containing diverse proteins that originate from both within and outside the eye.[56, 57] The finding of retinal proteins in the vitreous suggests that vitreous biopsies could be used as an indirect method for molecular biopsy of the retina. We would expect that retina-specific biomarkers would be highly elevated in degenerative retinal diseases, and our data set will allow further investigation into this concept.[18] We are just beginning to understand the molecular constituents of the vitreous in health and disease, and translational proteomics may more effectively direct efforts to cure blindness.

## Supporting Information

S1 Table Human vitreous proteins table. Data from the tandem mass spectrometry provides the relative expression levels for all the individual proteins isolated from the anterior hyaloid (AH), vitreous cortex (VC), vitreous base (VB) and vitreous core (core).
(XLS)

S2 Table Venn Diagram subunits. (XLS)

S3 Table Enrichment analysis report for total vitreous proteins by pathway. (XLS)

S4 Table Enrichment analysis report for proteins unique to the anterior hyaloid. (XLS)

S5 Table Enrichment analysis report for proteins unique to the vitreous cortex. (XLS)

S6 Table Enrichment analysis report for proteins unique to the vitreous base. (XLS)

S7 Table Enrichment analysis report for proteins unique to the vitreous core. (XLS)

S8 Table Heatmap protein lists. (XLS)

S9 Table DAMPs. (XLS)
